# *Leptospira interrogans* in mammals in Lviv Oblast, Ukraine, 2001-2015

**DOI:** 10.1371/journal.pntd.0007793

**Published:** 2019-12-02

**Authors:** Olena Zubach, Oksana Semenyshyn, Lesya Hatsji, Mykhaylo Demchyshyn, Aleksander Zinchuk

**Affiliations:** 1 Danylo Halytsky Lviv National Medical University, Lviv, Ukraine; 2 State Institution Lviv Oblast Laboratory Center of the Ministry of Health of Ukraine, Lviv, Ukraine; 3 Chief Administration of the State Service of Ukraine on Food Safety and Consumer Protection in Lviv Oblast, Lviv, Ukraine; University of Connecticut Health Center, UNITED STATES

## Abstract

This study describes changes in the prevalence of *Leptospira interrogans* infections among small mammals, including rats and larger domestic and wild mammals in Lviv Oblast, a region in western Ukraine from 2001–2015, using the microscopic agglutination test (MAT). A total of 439,948 domestic or wild animals were tested. We found the prevalence of *Leptospira interrogans* exposure varied among tested species and changed over the time. Infection was significantly less common in domestic animals, than in wild rodents. In swine the overall seroprevalence was 0.51%, while in cattle it was 0.19%. In dogs it was higher—2.75%. After 2006, evidence of infection was only observed in swine among domestic animals. The prevalence among large wild animals (0.25%) was similar to that among domestic animals. Among small mammals and rats, seroprevalence was most commonly observed among *Rattus norvegicus* (18.44%) and it was less common among other wild small mammals (8.74%). There were two dominant serogroups among large wild and domestic animals–*L*. *icterohaemorrhagiae* and *L*. *hebdomadis* while among wild small mammals the two most common were *L*. *icterohaemorrhagiae* and *L*. *grippotyphosa*. Wild animals with antibodies were found throughout the entire oblast.

## Author summary

Leptospirosis is one of the most prevalent bacterial zoonoses in the world. The main reservoir of the disease is rodents. Fur animals, domestic animals and livestock are an additional reservoir and also pose a threat of infection for humans. Therefore, the profound interest to *Leptospira* infection among the main and secondary reservoirs is important considering the direct threat for humans to get infection from these animals. We analyzed the official annual reports of the Lviv Regional State Laboratory of Veterinary Medicine of the Chief Administration of the State Service of Ukraine on Food Safety and Consumer Protection in the Lviv Oblast and the Laboratories of Especially Dangerous Infections of the Lviv Oblast Laboratory Center of the Ministry of Health of Ukraine for the period of 2001–2015 with regard to *Leptospira* infection rate in rodents, domestic animals and livestock, as well as wild animals in Lviv Oblast, Ukraine (Eastern Europe). During this period, no leptospirosis cases was recorded in cattle and horses. Cattle were infected only in 0.19% of samples, and *Leptospira hebdomadis* was identified in all cases. In contrast, all cases of infection among pigs (0.51%) and dogs (2.75%), as well as wild fauna (0.25%) were caused by *L*. *icterohaemorrhagiae*. Infection rates in rodents during the study period reached 8.74%, with *L*. *grippotyphosa* detected the most frequently (85.94%). While testing rats, we determined that almost every fifth rat (18.44%) was infected by *Leptospira*, and *L*. *icterohaemorrhagiae* was found the most frequently (71.83%). The results reveal that the main preventive measure against the potential threat of leptospirosis in humans is connected to eliminating the significant level of infection in rats and other rodents. Therefore, active deratization should remain a priority in the prevention of leptospirosis in Lviv Oblast of Ukraine.

## Introduction

*Leptospira interrogans* is a bacterial pathogen that infects numerous species of mammals, causing disease in both people and domestic animals globally. It includes numerous serotypes that tend to occur predominantly in small subsets of species [1]. In Ukraine, surveillance for *L*. *interrogans* serogroups, in animals, is performed annually as part of the responsibilities on the State and National government. Collected samples are screened serologically for evidence of recent infection and reported by species. This surveillance is performed because leptospirosis in humans is relatively common, and a nationally reportable disease [2, 3]. For example, there were 323 cases of the disease in humans registered in 2016, and 332 cases in 2017.

Lviv Oblast is located in the western part of Ukraine and is adjacent to the eastern Polish border and serves as an international ground transport route to Eastern Europe. It is a predominantly rural and agricultural area with a human population of 2,534,000. Of these, 989,300 people (39%) live in villages, while 29% live in the capital, Lviv [4]. The farmed agricultural land is 657,000 hectares, or 59% of the total region [5]. Agricultural and forestry occupation is likely to lead to contact with animal reservoirs. In the region in 2016 and 2017, 14 cases, and 18 cases of leptospirosis were diagnosed, respectively [6].

In this study, we describe *L*. *interrogans* changes in patterns of exposures among mammals surveyed during surveillance efforts in 15 years (2001–2015).

## Materials and methods

### Collection of mammals

#### A total of 439,948 domestic or wild animals were tested

According of State Sanitary Rules of the Ministry of Health of Ukraine, the institutions of the State Sanitary and Epidemiological Service of Ukraine must collect, transport, and examine wild animals and perform surveillance in natural foci of infectious diseases, such as leptospirosis. As standard surveillance, specimens of wild small mammals and rats were collected in all 20 administrative districts (raions) of Lviv Oblast.

The mammals were collected from February to October each year. Small mammals and rats were trapped using wooden live traps, in forest biotopes and on the borders of forest plots and agricultural areas. They were euthanized under anesthesia, and 1 ml blood samples were collected from the hearts using sterile pipettes, then centrifuged to separate sera. Samples collected from small mammals and rats were stored at 2–8°C (up to 24 hours) or at –20 °C (over 24 hours) [7].

Sample collections from domestic and larger wild animals were performed in compliance with the Instruction for Laboratory Diagnostics of Leptospirosis in Animals [8]. Blood samples (5 ml) were collected from animals’ veins with sterile syringes; then the blood was centrifuged, and serum was separated; before testing, the serum was stored at 2–8 °C (up to 24 hours) or -20 °C (longer than 24 hours). Serum samples from pigs, cattle, small ruminants, and horses from breeding farms that were imported or exported to and from Lviv Oblast were required to be tested. Additionally, cattle from private households and farms were routinely tested. Sera from kennel dogs and pet dogs (brought for veterinary examination by their owners, as well as military service dogs) were obtained and tested using methods described above. Blood samples also were obtained from wild animals collected by gamekeepers during regular culling, using similar methods.

### Serological testing

Serological testing of small mammals was performed at the Especially Dangerous Pathogens (EDP) Laboratory of Lviv Oblast Laboratory Centre of Ministry of Health of Ukraine, while testing of domestic animals and wild fauna was performed at the Lviv Regional State Veterinary Laboratory.

Microagglutination tests (MAT) were performed for sera from collected animals using 15 serovars serogroups: *L*. *cabura*, *L*. *serjoe*. *L*. *icterohaemorrhagiae*, *L*. *javanica*, *L*. *canicola*, *L*. *autumnalis*, *L*. *australis*, *L*. *pomona*, *L*. *grippotyphosa*, *L*. *bataviae*, *L*. *tarassovi*, *L*. *hebdomadis*, *L*. *pyrogenes*, *L*. *ballum*, *L*. *сynopteri* for small mammals; and 9 serogroups: *L*. *icterohaemorrhagiae*, *L*. *grippotyphosa*, *L*. *canicola*, *L*. *tarasovi*, *L*. *pomona*, *L*. *kabura*, *L*. *polonica*, *L*. *hebdomadis* and *L*. *bratislava*. Sera were initially screened for *Leptospira* spp. antibodies, at dilutions of 1:50 for domestic animals and 1:10 for wild animals, according to standard operating procedures.

The MAT was performed using standard microtiter methodology [8]. Antigens consisted of live 4-day cultures of the desired *Leptospira* strain standardized to a density of 100 or more organisms per field in dark-field microscopy with good mobility, not showing spontaneous agglutination or foreign particles (e.g., precipitate).

Blood serum was inactivated at 65 °C for 30 min and then diluted with normal saline (pH 7.2–7.4) starting at 1:10 [9]. Diagnostic cultures were added to wells of polystyrene plates, then one drop of sample serum was added to the appropriate wells. The plates were covered to prevent evaporation and were incubated for 1 hour at 37 °C. The mixture of *Leptospira* cultures with normal saline at 1:1 was used as a blank control.

The resulting preparation "crushed drop" was considered using dark field microscopy. The standard preparation glasses and 15x15 mm cover glasses were used. Microagglutination was visualized as *Leptospira* “gluing” and as formation of “spiders”, “bows”, and “braids.” The degrees of agglutination were recorded as 1+ with 25% of the leptospires clumped, 2+ with about 50% clumped, 3+ with approximately 75% clumped, and 4+ when 75 and 100% agglutination occurred [8,9]. The end point was taken as the highest dilution, showing a 2+ reaction. The results were valid only in the absence of any lysis and agglutination in the control. Positive and negative control sera from high tittered and healthy animals, respectively, were used each time the test was performed.

### Leptospira cultivation

To obtain direct evidence of the causative agent in the region, bacteriological studies to isolate live cultures from the environment or host species (rats or other rodents) were performed in parallel with serological testing. Terskikh selective liquid medium with 2.5% of inactivated rabbit blood sera was used for *Leptospira* isolation from rodents. Rodent biological material (adrenal cortex) was inoculated into the medium and incubated at 28 °С for 28–30 days; every 5–7 days, we checked it in dark-field microscopy. If sufficient numbers (50–100 per field) of alive, mobile microorganisms were observed, we inoculated the isolate to new media for further accumulation of biological mass to identify features and perform serotyping with commercial kits of diagnostic specific sera (Armaviskaya Biological Factory, Russian Federations) in MAT.

### Mapping

We mapped the distributions of infected rodents using the nearest town or village as the geographic site for collection. The map was created using Quantum GIS (QGIS version 2.18) based on the data described in the paper. The layers with administrative boundaries were downloaded from the Database of Global Administrative Areas (GADM, https://gadm.org/about.html).

### Statistical methods

Data about all animals was recorded in a single Excel database. Statistical analysis was conducted using Chi-square tests.

### Ethical considerations

This study was granted an exemption from reviewers at the Institutional Review Board of SI “Public Health Center MoH of Ukraine” (registration # 00011557, identification number of expertise IRB2019-18).

Our study provides the results of the retrospective analysis of the official reports from the state regional veterinary and public health authorities. No actual sample collection was performed by the authors.

Wild and domestic animals were tested for leptospirosis in the framework of routine laboratory surveillance implemented by the Lviv Regional State Veterinary Laboratory. These studies were conducted as a part of the state monitoring for animal leptospirosis in the area. The collection of blood samples from wild and domestic animals was carried out in compliance with bioethical principles. Representatives of veterinary authorities received verbal consent for sampling and testing from the owners of domestic animals and livestock.

Collection and testing of gray rats and other rodents was carried out by specialists of the Laboratory of Especially Dangerous Infectious Diseases of the Lviv Oblast Laboratory Center of the Ministry of Health of Ukraine as part of monitoring for natural foci of leptospirosis. The studies are regulated by the current legislation in Ukraine (orders of the Ministry of Health of Ukraine). Rodents were caught in live traps, and then they were euthanized using chloroform or ether. Samples of blood (serum) and other organs were taken for testing posthumously. The procedures for the capture and sample collection from rodents were performed in accordance with the CDC’s recommendations [10].

## Results

Between 2001 and 2015, a total of 424,657 domestic animals of five species were tested by MAT (Table 1). The majority (99.2%) were agricultural species, primarily swine and cattle. Additional 3,912 individuals of larger wildlife were opportunistically collected. These included 462 (11.8%) rabbits, 376 (9.61%) foxes, 1627 (41.59%) wild pigs and 52 (1.33%) wild boars, 55 (1.4%) roes, 43 (1.1%) wolves, 1068 (27.3%) wild goats, 135 (3.45%) Guinea pigs, one (0.03%) bison, one (0.03%) lama, one (0.03%) marten, 45 (1.15%) wild dogs, 34 (0.87%) deer and 12 (0.69%) individuals were not identified to species in the reports.

**Table 1:** Number of tested and seropositive large domestic and wild animals*.

Animal species	Number of examined animals	Number of seropositive animals	
		Absolute value	%
Pigs	174,292	888—*L*.*icterohaemorrhagiae*	0.51
Cattle	234,147	445—*L*. *hebdomadis*	0.19
Small ruminants	5,989	0	0
Horses	6,665	0	0
Dogs	3,564	98—*L*.*icterohaemorrhagiae*	2.75
Wildlife	3,912	10—*L*.*icterohaemorrhagiae*	0.25

*—Table does not include rats or other small wildlife

According to the Lviv Regional State Veterinary Laboratory data, results of MAT demonstrated substantial differences in the prevalence of seropositive individuals among different species of animals. Dogs (2.75%; N = 98) were the most commonly positive large mammal, and in all cases *L*. *icterohaemorrhagiae* was the identified etiological agent. Domestic dogs were most commonly positive early in the study (2001–2008). In the latter portion of the study, no seropositive dogs were found even though 1,417 dogs were tested. Domestic pigs also were infected (0.51%; N = 888) with *L*. *icterohaemorrhagiae* but significantly less often (X^2^ = 301.65; p < 0.001) than dogs. Despite the lower prevalence, the substantially larger numbers of swine led to substantially larger total numbers of positive tests. Unlike canines, positive swine were found throughout the duration of the study. Among cattle (N = 60,450 animals tested), 445 positive individuals (0.19%) were found infected by *L*. *hebdomadis*. All these positive cattle were observed between 2001 and 2006. Ten individuals (0.25%) among larger wildlife species (N = 3912) were positive to *L*. *icterohaemorrhagiae*. All ten were observed in 2003, and included one wild boar and nine wild dogs. Antibodies were not detected by MAT in the small ruminants (N = 5989) or horses (N = 6665).

A total of 11,379 individual small mammals were collected for testing (1790 rats and 9589 other small mammals). There were 1169 MAT positive individuals (overall prevalence = 10.3%). As was observed with domestic animals, there was substantial variation in the prevalence among small mammals, both across species and within species across years. Two predominant serogroups circulated among small mammals, although other serogroups were occasionally reported.

**Fig 1:**
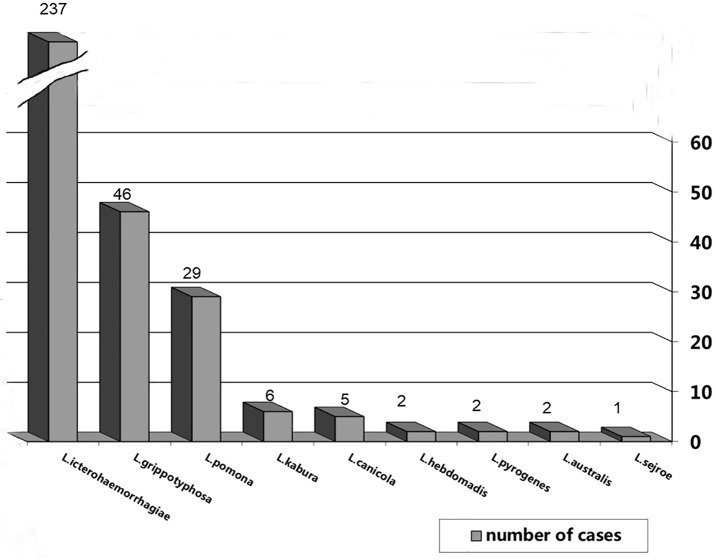
Serogroup belonging of leptospira in seropositive rats with using of MAT (n = 330).

*Rattus norvegicus* was the most commonly positive species (18.44%; N = 330). It also had the most diverse array of identified serogroups. Overall, most *R*. *norvegicus* were exposed to *L*. *icterohaemorrhagiae* (237/330; 71.83%). This serogroup, detected by MAT, was reported during every year of the study. Serogroup *L*. *grippotyphosa*, in 46 animals (13.94%), *L*. *pomona* in 29 (8,79%), and *L*. *kabura*–in 6 (1.8%), were also observed in more than one year of the study. Serogroup *L*. *canicola* in 5 (1.5%), *L*. *hebdomadis*, *L*. *pyrogenes* and *L*. *australis* each in 2 (0.61%) cases and *L*. *sejro* in 1 (0.3%) rat were only observed during single years (Fig 1).

**Fig 2:**
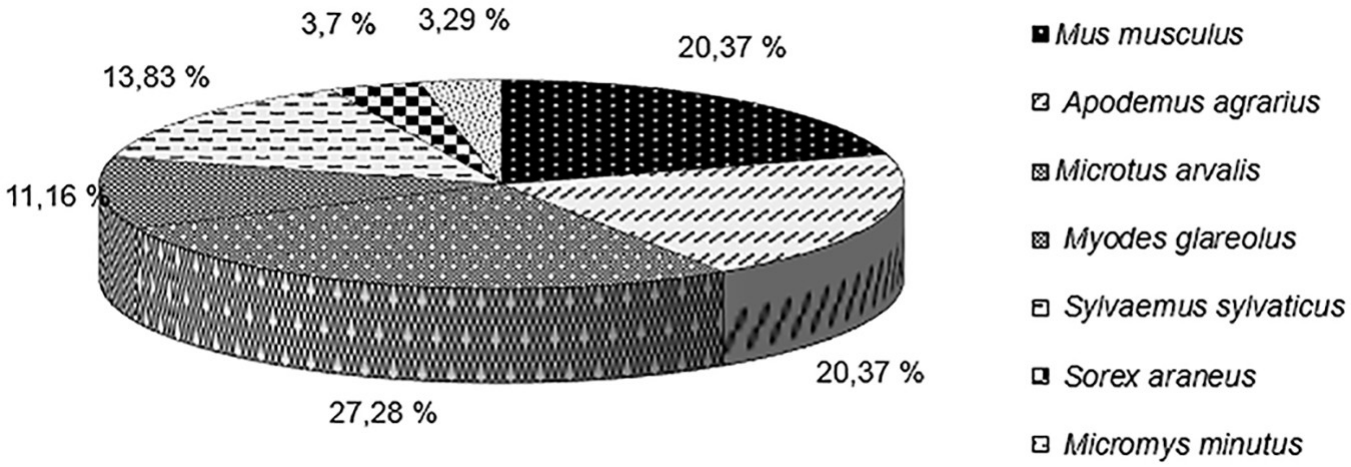
Species composition of sampled small mammals (n = 9,589).

In Lviv Oblast, the sampled small mammal fauna in natural biotopes were from the order of *Rodentia* and *Insectivora*. In different natural climatic landscapes (mixed forest, forest-steppe, Carpathian mountains), the species composition and abundances of rodents differed. Seven rodent species dominated, including: *Microtus arvalis* (27.28%), *Mus musculus* (20.37%), *Apodemus agrarius* (20.37%), *Sylvaemus sylvaticus* (13.83%), *Myodes glareolus* (11.16%), *Sorex araneus* (3.7%), *Micromys minutus* (3.29%) (Fig 2). Abundances of these rodent species varied across years accounting from 7.0 to 9.9 per 100 trap-days. The highest abundances occurred during summer and autumn periods.

**Fig 3:**
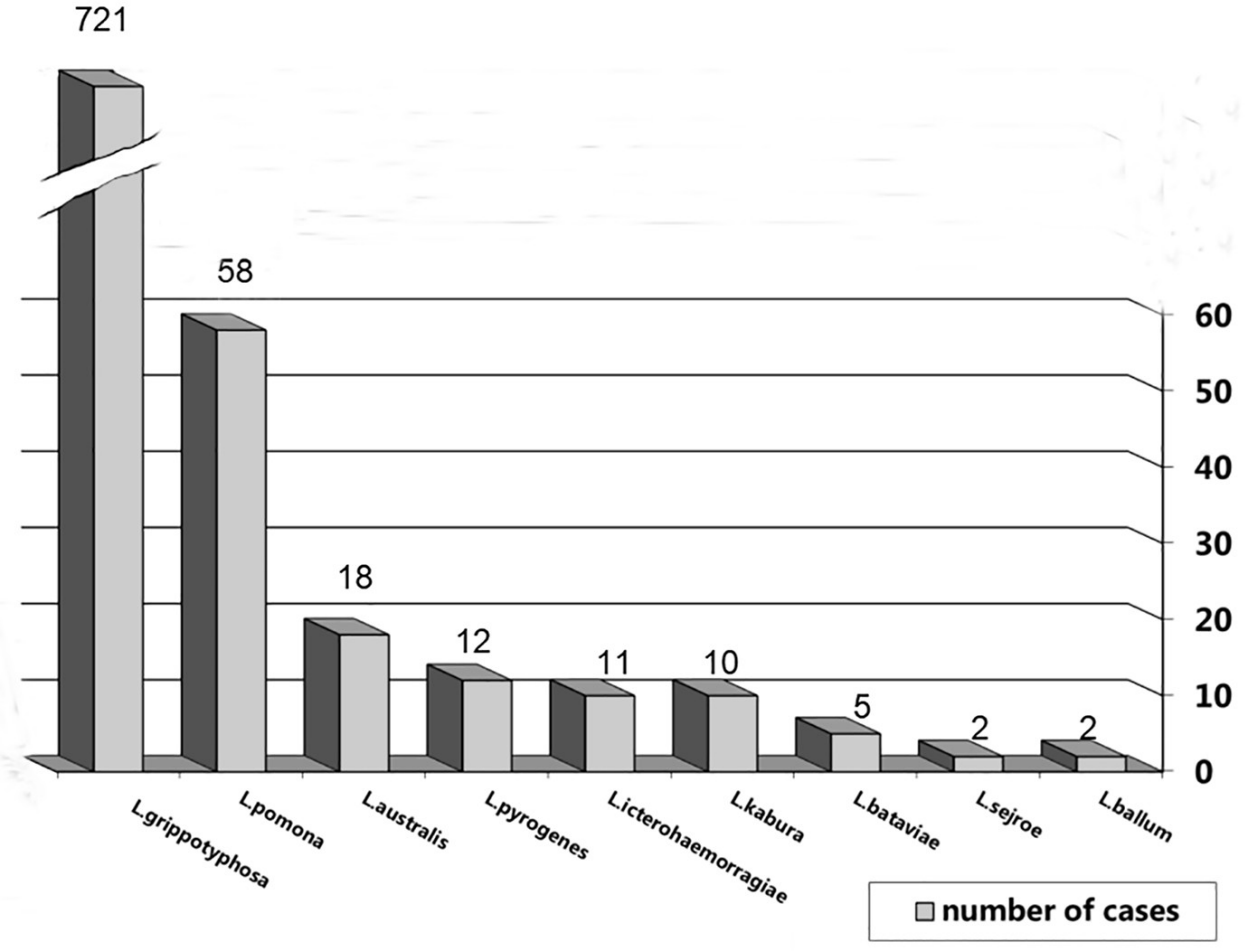
Serogroups of leptospirae in small mammals (excluding *R*. *norvegicus*) using of MAT (n = 839).

Antibodies to *L*. *grippotyphosa* were detected in 721 (85.94% of the positive animals) individuals from the seven species of small mammals. Antibodies to *L*. *pomona* were detected in 58 (6.91%) individuals, in five species of small mammals. Antibodies were less frequently detected to *L*. *australlis* in 18 (2.15%) individuals, *L*. *pyrogenes* in 12 (1.43%) individuals, *L*. *kabura* in 10 (1.19%) individuals, *L*. *bataviae* in 5 (0.60%) individuals, *L*. *sejro* and *L*. *ballum–* 2 individuals each (0.24%) (Fig 3).

Among seven species of small mammals, *Leptospira* prevalence and etiological structure for each species differed significantly (X^2^ = 240.87, 6df; p < 0.0001). The highest seroprevalence was found in *Microtus arvalis* (15.94%) and was significantly elevated compared with the remaining six species (Multiple Comparison for Proportions Critical value Q = 4.882) The remaining species did not differ significantly from one another (Table 2).

**Table 2:** *Leptospira spp*. prevalence among species of small mammals.

Species of small mammals	Total	Positive		Negative	
		absolute number	%	absolute number	%
*Mus musculus*	1,953	99	5.07	1,854	94.93
*Apodemus agrarius*	1,953	140	7.17	1,813	92.83
*Microtus arvalis*	2,616	417	15.94	2,199	84.06
*Myodes glareolus*	1,070	69	6.45	1,001	93.55
*Sylvaemus sylvaticus*	1,326	71	5.35	1,255	94.65
*Sorex araneus*	355	27	7.61	328	92.39
*Micromys minutus*	316	16	5.06	300	94.94
Total	9,589	839	100	8,750	100

**Fig 4:**
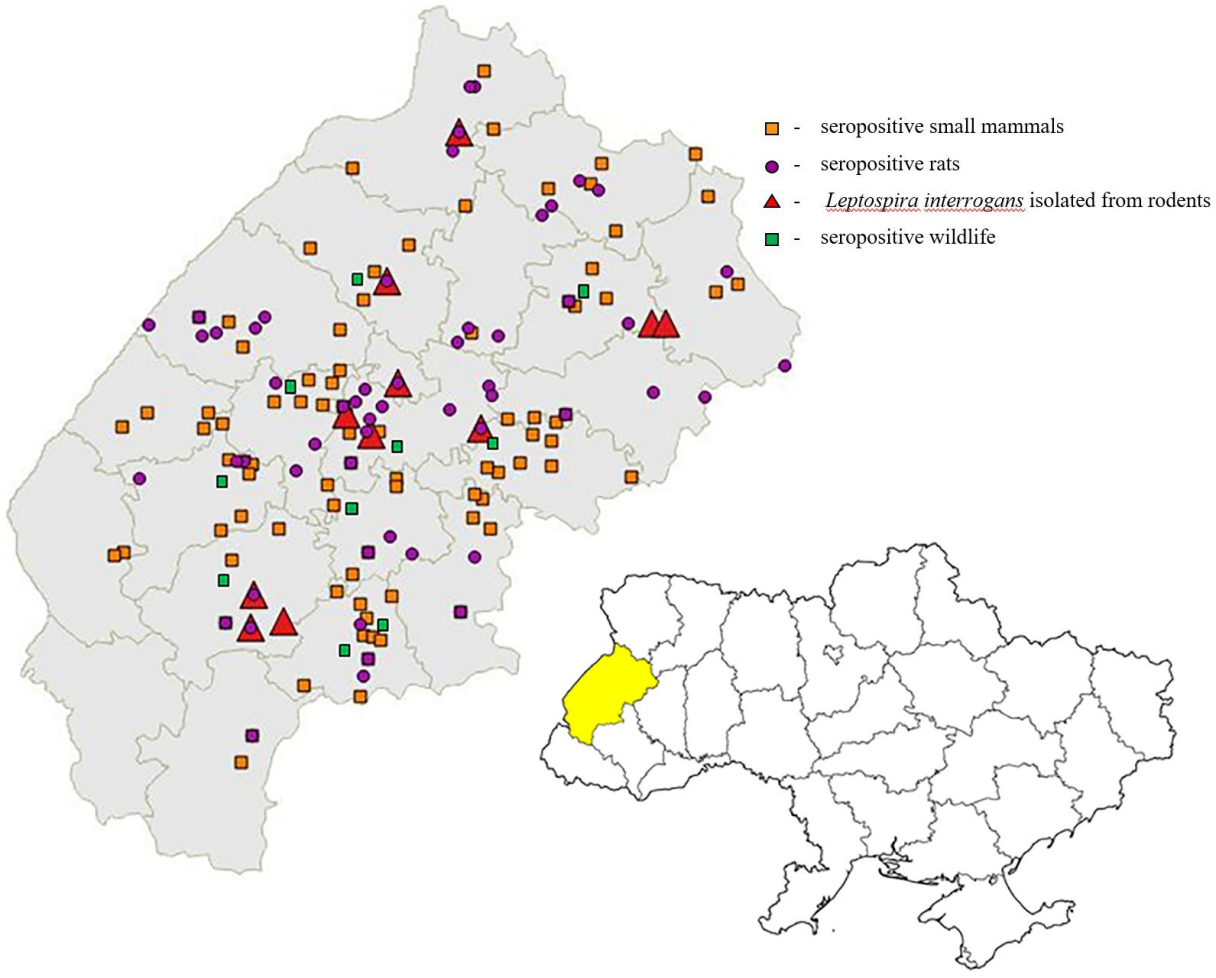
Map of distribution of wildlife animals and rodents infected of Leptospira interrogans*. *The Figure 4 was created using Quantum GIS (QGIS version 2.18); the layers with administrative boundaries were downloaded from the Database of Global Administrative Areas (GADM, https://gadm.org/about.html), please see the license: https://gadm.org/license.html.

At the town/village level, seropositive rats and mice were found throughout the Oblast (Fig 4). Positive animals were collected from 192 towns and villages (91 locations were associated with *R*. *norvegicus* while 111 towns/villages yielded other species of infected small mammals). *L*. *icterohaemorrhagiae* (44 cultures) was attempted from a subset of available materials and isolations were made from small mammals collected in 14 towns and villages (Fig 4).

## Discussion

Based on serological monitoring of *L*. *interrogans* in domestic and wild animals in Lviv Oblast we find substantial variation in the prevalence of infection depending on species and serogroups over the 15 years study. For most of the domestic species there was a decreasing trend in infection, over time. This was most evident in dogs and cattle. In both species, infection was absent during the last decade of the study. In others, such as horses and small ruminants, infection was never detected. There also was substantial variation in serogroup types depending on species. For example, *L*. *hebdomadis* was typically found in cattle prior to 2006, while *L*. *icterohaemorrhagiae* was observed in *R*. *norvegicus*, swine, and domestic dogs. The other serogroups were found only in the various small mammal species, and then many were only seen for one or two years (Fig 3).

The results suggest that *L*. *icterohaemorrhagiae* exposure was prevalent among wild boars and domestic pigs. Similar studies in neighboring Poland showed a wider range of *Leptospira* serogroups detected in these animals, namely *L*. *ballum*, *L*. *sejroe*, *L*. *pomona*, *L*. *Icterohaemorrhagiae* [11,12]. Polish researchers also reported detection of antibodies to *L*. *bratislava* [13] in horses and dogs; and *L*. *grippotyphosa* and *L*. *pomona* [14] among wild deer. In Croatia, *L*. *grippotyphosa* [15] was most common in wild boars.

In our historical study, the highest overall seroprevalence among domestic animals was found in dogs (2.75%). However, there was a sharp decline in infection during the latter half of the study when more than 1,400 animals were tested but no positive animals were observed. The other most common serogroup, *L*. *hebdomadis*, was occasionally observed in cattle (0.19%). However, as with dogs, infection was eliminated from the surveyed population and not detected after 2006. No infections were ever observed in horses or small ruminants throughout the study. Swine (0.51%) appeared to be the only domestic species that remained infected in this study.

Among wild animals, exposure was prevalent in two species, *R*. *norvegicus* (18.44%) and *M*. *arvalis* (15.94%). In addition to *L*. *icterohaemorrhagiae* in *R*. *norvegicus*, *L*. *grippotyphosa* was predominant in *M*. *arvalis*, with less frequent occurrences in other small mammals. The wild small mammal community structure changed during our study. In the beginning (2001–2003), *Myodes glareolus* (25.55%), *M*. *arvalis* (31.95%) and *A*. *agrarius* (17.19%) were the most abundant species sampled. This shifted by the end of the study (2013–2015). *Microtus arvalis* (29.0%) remained dominant but *Mus musculus* (26.21%) had become co-dominant.

Throughout the study, *L*. *grippotyphosa* (75.0–100.0%) and *L*. *pomona* (3.60–20.0%) were most abundant among small mammals, however, in later years, other serovars also were reported so that *L*. *bataviae* (9.89–15.0%) and *L*. *australis* (in 2015–40.0%) also were evident. Similar results of *Leptospira* serological spectrum in small mammals were obtained in Zakarpattia Oblast of Ukraine [16], as well as in other countries where climate and landscape are close to conditions in Ukraine. As an example, *L*. *grippotyphosa* [17] was the most commonly found in MAT in Czech Republic, while in Croatia, *L*. *pomona*, *L*. *serjoe*, or *L*. *australis* [15] were found in small mammals.

Major advantages of this study include the long period of monitoring (15 years) and large sample sizes of domestic and wild animals (in total, 439,948 individuals were studied). This allowed us to document the temporal dynamics of *Leptospira* occurrence in the region. This included the decline of detected exposure to zero in most domestic species (except swine, where it is remains uncommon) and to observe changes in community structure and serogroups over time. A total of three *Leptospira* serogroups were detected most frequently, with *L*. *icterohaemorrhagiae* and *L*. *hebdomadis* primarily occurring in two species of large wild and domestic animals, and *L*. *icterohaemorrhagiae* and *L*. *grippotyphosa* predominating among two species of small wild mammals.

The obtained data demonstrate changes in *Leptospira* species composition and serogroup structure over the past 15 years, but the reason for this change is a subject for further studies. Our hypotheses include possible change of habitat areas of specific species of small mammals in the territory of the Lviv region that might be caused by both climate and landscape changes, in particular, temperature rise, and active economic activities (intensive deforestation, agromelioration and agricultural work, progressive building works in natural biotopes). Combination of these factors contributes to the forced migration of small mammals and consequently the change of species dominants.

Another advantage of this study is that all tests were performed using standardized methods within the same laboratories. Serological surveys for *Leptospira* using MAT generally have a relatively high specificity and sensitivity [8].

Thus, the 3 serogroups mentioned above are predominant among *Leptospira interrogans* in Lviv Oblast and act as main causative agents in leptospirosis incidence among animals and represent the largest threats to humans and domestic animals at this time.

Limitations of the study included its retrospective nature that led to use of previously recorded information about animals, reflected only in available official annual reports of responsible institutions. We also cannot exclude the possibility of cross-reactions in MAT with other spirochetes. Another limitation of the study was different numbers of animals of each species in the groups.
